# Chronic Enteroviral Meningoencephalitis in a Patient with Good’s Syndrome Treated with Pocapavir

**DOI:** 10.1007/s10875-022-01321-6

**Published:** 2022-07-23

**Authors:** Alexandros Grammatikos, Philip Bright, Justin Pearson, Marcus Likeman, Mark Gompels

**Affiliations:** North Bristol NHS Trust, Bristol, UK

To the Editor,

Enteroviruses are a diverse selection of viruses which include polioviruses, coxsackieviruses, echoviruses, and others. Severe or persistent enteroviral infection is well-documented in patients with antibody deficiency, mostly in those with X-linked agammaglobulinemia (XLA) or following anti-CD20 monoclonal antibody therapy [1]. Here, we report the first case of chronic enteroviral meningoencephalitis (EME) in a patient with Good’s syndrome and discuss potential treatment options.

## Description

This male patient presented, aged 27, with recurrent chest infections and weight loss. Two years later, a diagnosis of cortical thymoma (type B2) was made, and he underwent a successful surgical excision. Post-operatively, he continued to have multiple infections, mainly pneumonias, and investigations revealed low serum immunoglobulins (Supplementary Table 1). He was referred to Clinical Immunology where a complete absence of peripheral B cells was identified, with minimal bronchiectasis on a thoracic CT scan. The combination of thymoma with immunodeficiency led to a diagnosis of Good’s syndrome, and he was commenced on immunoglobulin replacement, with prophylactic co-trimoxazole, to good effect.

Aged 36, he suffered a brief episode of right hemiparesis with dysesthesia and dysarthria. Brain MRI, CT head, echocardiogram, and carotid doppler did not reveal a cause for his symptoms, and these were attributed to a transient ischemic attack. Over the following years, he attended the emergency department three more times for neurological symptoms of vertigo, ataxia, dizziness, and/or facial dysesthesia, with continuing normal brain imaging.

**Fig. 1:**
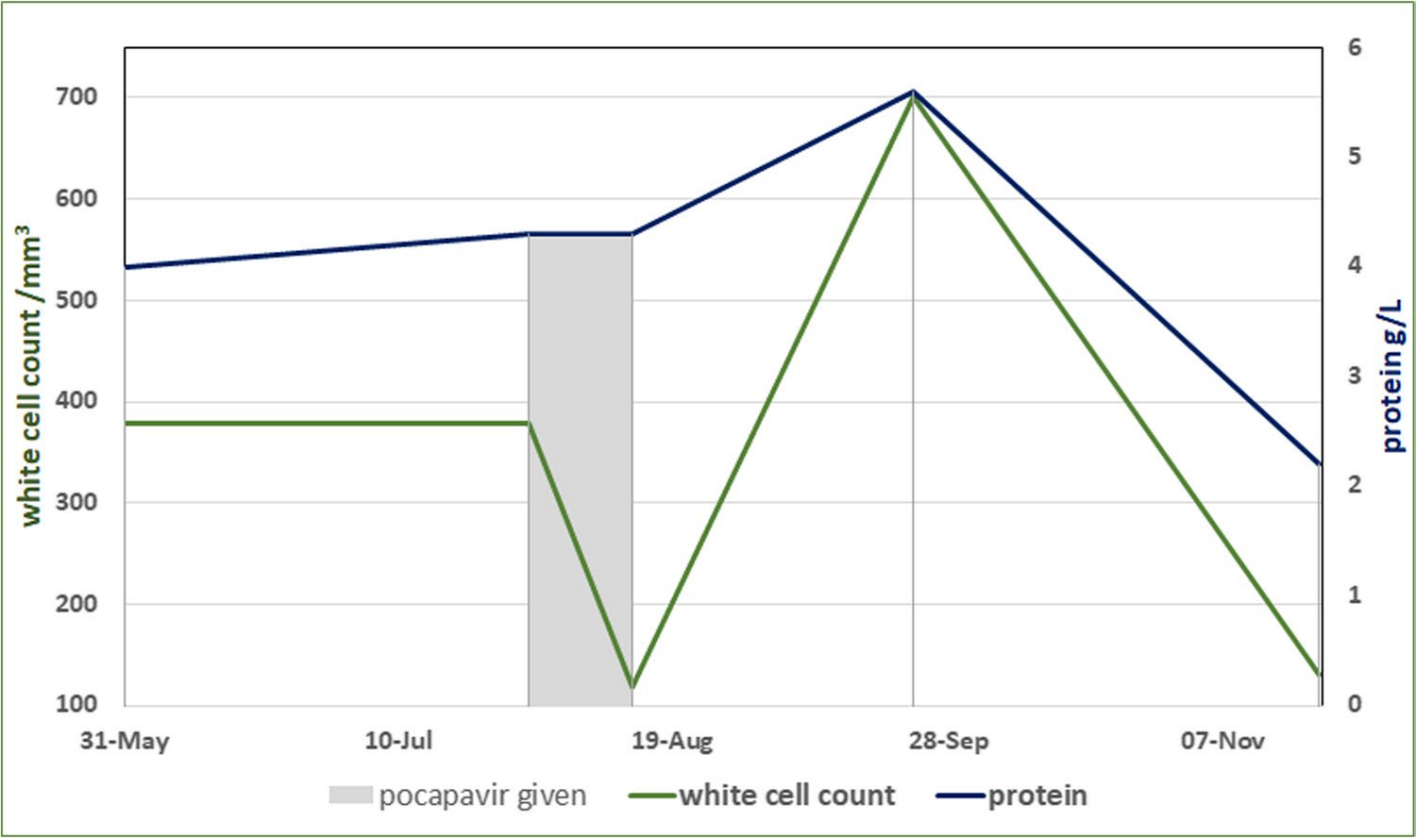
Progression of the patient’s cerebrospinal fluid laboratory parameters over time (white cell reference range < 1/mm.^3^, protein reference range < 0.61 g/L)

Aged 41, he presented with an episode of facial and left upper limb dysesthesia. A CT head was normal, but an MRI at that stage revealed white matter high T2 intensity suggestive of transependymal edema. Over the next 2 months, he complained of increasing headaches with photophobia and was found to have a mild cognitive impairment (23/30 on Montreal Cognitive Assessment test). He was later admitted to hospital with a focal seizure, left facial weakness, and dysarthria. An EEG revealed abnormal asymmetrical activity in his frontal lobe, but no seizure activity. His repeat MRI revealed progression of the previously noted abnormalities, with an enlargement of the ventricular system in keeping with hydrocephalus and a rarely described pattern of T2 high intensity in the claustrum (Supplementary Fig. 1). A subsequent CT head confirmed communicating hydrocephalus (Supplementary Fig. 1 and Supplementary Table 2), and a lumbar puncture revealed a significantly raised cerebrospinal fluid (CSF) protein (Fig. 1). CSF negative for oligoclonal bands, but this may have been inaccurate in view of the antibody deficiency. CSF was 60% lymphocytic and enterovirus type D68 was identified by PCR. This was confirmed in further lumbar punctures, but his blood, throat, and stool samples remained consistently negative for enterovirus (Supplementary Table 3). His IgG trough on replacement immunoglobulin at that stage was good, > 8 g/L.

He was treated with levetiracetam and an experimental drug for enteroviral infection, oral pocapavir (MedChemExpress LLC) at a dose of 1600 mg once daily for 14 days, which was tolerated well. His immunoglobulin dose was also increased, and an external ventricular drain was inserted to relieve the hydrocephalus. He had a fluctuating course, initially responding to this treatment with improvement of his clinical and CSF laboratory picture (Fig. 1), but soon relapsed. After a prolonged hospital stay, he made a partial recovery but remained positive for enterovirus in his CSF and was unable to drive or work for several years. Currently, at the age of 46, he has significantly recovered, managed to return to work and is able to drive but exhibits intermittent bilateral hand tremor. He tests negative for serum IgG antibodies to alpha, omega, and beta interferon (multiplexed particle-based flow array).

## Discussion

This is the first case report of chronic EME in Good’s syndrome (Supplementary methods) [1]. EME is however well-described in other immunodeficiencies, particularly XLA and following-B cell-depleting monoclonal antibody therapy. A systematic review of 101 EME cases found that more than a quarter of them suffered from XLA, while 20 had previously received B cell-depleting therapies [2]. Also, in a series of 90 patients with immunodeficiency and chronic EME, more than half had XLA and 20 had CVID [3]. The higher incidence of chronic EME in patients with low peripheral B cells suggests an important role for B cell immunity in the defense against this virus [1]. EME can also be seen in healthy individuals but is mostly short-lived and with a mild phenotype [4].

EME in immunodeficiency commonly presents with slowly progressive loss of motor and cognitive milestones, but also various symptoms like headaches, seizures, paresthesias, and vision loss. Patients can sometimes present with a dermatomyositis-like syndrome, before progressing to EME. EME has a high mortality in immunodeficiency, a previous study citing a 43% rate, usually within 2 years of diagnosis [3]. Of those who survive, many suffer long-term sequalae, like chronic infection and hydrocephalus, and only a few recover fully [3]. The risk of chronic EME is fortunately lower today, due to the widespread use of immunoglobulin therapy [4].

From early reports, it became clear that low serum IgG is a predisposing factor for EME and that immunoglobulin therapy, with serum IgG levels above 8 g/1, are protective. Immunoglobulin, administered IV or intrathecally, has also been trialed for the treatment of EME [2]. However, coverage for the various enteroviruses by immunoglobulin products is not universal, which may explain why this is often unsuccessful, and also why infections sometimes occur in patients that are on adequate IgG replacement therapy [4].

The only drug specific for EME, pleconaril, is unfortunately no longer available [4]. Many other drugs have been tried, e.g., ribavirin, cidofovir, fluoxetine, subcutaneous IFNα, and intrathecal IFNβ, with mostly poor outcomes [4]. Pocapavir, a capsid inhibitor that blocks virus uncoating and viral RNA release into cells, is another investigational drug candidate for enteroviruses. In vitro and animal models have revealed that it has potent antiviral activity against polio and non-polio enteroviruses. In humans, it has been shown to be effective in neonatal cases [5], but only very few reports on its use in adults have been published. Virological and clinical response to pocapavir treatment, in this case, was mixed, ultimately with a good but delayed outcome, making interpretation of efficacy difficult.

In conclusion, EME is an important diagnosis to consider in patients with antibody deficiency and neurological symptoms, and may be prevented by immunoglobulin replacement therapy. Prompt treatment is crucial to avoid the significant morbidity and mortality associated with this disease, and pocapavir offers a promising option for the clinician.

## Supplementary Information

Below is the link to the electronic supplementary material.

Supplementary file1 Supplementary Figure 1. Radiological investigations at the point of diagnosis of enteroviral meningoencephalitis: (a & b) CT head showing communicating hydrocephalus; (c & d) MRI head showing a rarely described pattern of T2 high intensity in the claustrum (arrows) and hypointensity in the lateral ventricles (JPG 57 KB)

Supplementary file2 (DOCX 23 KB)

## Data Availability

Not applicable.
