# Supplementary figures and images for: Chronic Enteroviral Meningoencephalitis in a Patient with Good’s Syndrome Treated with Pocapavir
Source: J Clin Immunol. 2022 Jul 23;42(8):1611–3. doi: 10.1007/s10875-022-01321-6 (PMC9700624; doi:10.1007/s10875-022-01321-6)

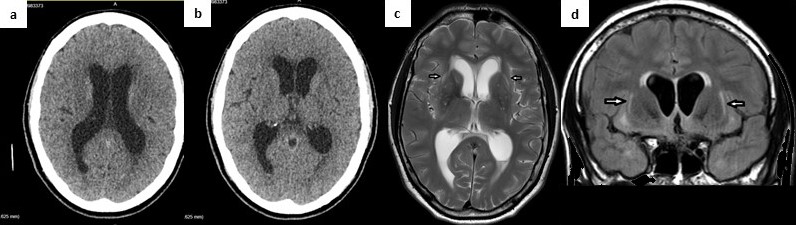

Supplement: Supplementary file 1 — Supplementary file1 Supplementary Figure 1. Radiological investigations at the point of diagnosis of enteroviral meningoencephalitis: (a & b) CT head showing communicating hydrocephalus; (c & d) MRI head showing a rarely described pattern of T2 high intensity in the claustrum (arrows) and hypointensity in the lateral ventricles (JPG 57 KB) [file 10875_2022_1321_MOESM1_ESM.jpg]
