# Supplementary material for: Chronic Enteroviral Meningoencephalitis in a Patient with Good’s Syndrome Treated with Pocapavir
Source: J Clin Immunol. 2022 Jul 23;42(8):1611–3. doi: 10.1007/s10875-022-01321-6 (PMC9700624; doi:10.1007/s10875-022-01321-6)
Supplement: Supplementary file 2 — Supplementary file2 (DOCX 23 KB) [file 10875_2022_1321_MOESM2_ESM.docx]

**Supplementary** **methods**

Literature search was performed in Medline and Embase databases using the terms “good* syndrome”, “thymoma with immunodeficiency”, “enterovi* meningitis or encephalitis”.

|  | **SERUM** | **Reference range** |
| --- | --- | --- |
| **IgG** | 2.5 | 6-16 g/L |
| **IgA** | 0.3 | 0.8-2.8 g/L |
| **IgM** | <0.18 | 0.5-1.9 g/L |
| **C3** | 1.63 | 0.8-2.1 g/L |
| **C4** | 0.31 | 0.15-0.5 g/L |
| **CH50** | 100% | >70% |
| **AP50** | 100% | >70% |
| **White cell count** | 10.28 | 4-11 *10^9^/L |
| **Neutrophils** | 6.35 | 1.5-8 *10^9^/L |
| **Lymphocytes** | 2.68 | 1-4 *10^9^/L |
| **Monocytes** | 0.65 | 0.2-1 *10^9^/L |
| **T cells** | 2.49 (93%^*^) | 0.67-3.04 *10^9^/L |
| **CD4 T cells** | 1.53 (57%^**^) | 0.38-1.84 *10^9^/L |
| **CD8 T cells** | 0.83 (31%^**^) | 0.31-1.6 *10^9^/L |
| **NK cells** | 0.11 (4%^*^) | 0.1-0.76 *10^9^/L |
| **B cells** | <0.03 (<1%^*^) | 0.11-0.64 *10^9^/L |
| **Anti-acetylcholine**  **receptor** antibodies | <0.5 | <0.5 IU/ml |

**Supplementary** **Table 1.** Patient’s immunological investigations at the time of diagnosis (*of total lymphocytes, ** of total T cells).

| **Date** | **MRI findings** |
| --- | --- |
| J July | communicating hydrocephalus, all ventricles |
| 11 July | right frontal external ventricular drainage inserted  ventricular system improved |
| 18 July | external ventricular drainage removed  worsening hydrocephalus |
| 23 July-  6 August | Patient received pocapavir |
| 18 August | hydrocephalus slightly worsened, more dilatation of temporal and frontal horns, slightly increased sulcal effacement superiorly particularly over the left hemisphere |
| 13 September | slight deterioration of hydrocephalus with more prominent transependymal oedema |
| 30 September | ommaya reservoir inserted  slight reduction in calibre of the entire ventricular system |
| 23 November | some improvement of hydrocephalus |
| 29 November | reduction in size of ventricles and slender subdural effusions overlying both convexities and the cerebellum |

**Supplementary** **Table 2.** Progression of the patient’s hydrocephalus radiologically over time.

| **Date** | **Cerebrospinal fluid** | **Blood** | **Throat** | **Stool** |
| --- | --- | --- | --- | --- |
| 31 May | Positive |  |  |  |
| 2 June |  | Negative | Negative |  |
| 13 June | Positive |  |  |  |
| 8 July | Negative |  | Negative |  |
| 14 July | Negative |  |  | Negative |
| 20 July | Positive |  |  |  |
| 29 July | Positive low level | Negative |  |  |
| 10 August | Positive |  |  | Negative |
| 14 September | Positive |  |  |  |
| 23 September | Positive |  |  |  |
| 14 October | Positive low level |  |  |  |
| 31 October | Positive |  |  |  |
| 7 November | Positive |  |  |  |
| 14 November | Positive |  |  |  |
| 21 November | Positive low level |  |  |  |

**Supplementary** **Table 3.** Enterovirus testing in the patient’s cerebrospinal fluid, blood, throat and stool over time (patient received pocapavir from 23 July until 6 August).
